# Examining healthcare professionals’ beliefs and actions regarding the physical health of people with schizophrenia

**DOI:** 10.1186/s12913-020-05654-z

**Published:** 2020-08-20

**Authors:** Alexandra Berry, Richard J. Drake, Alison R. Yung

**Affiliations:** 1 Division of Psychology & Mental Health, School of Health Sciences, Faculty of Biology, Medicine and Health, University of Manchester, Manchester Academic Health Sciences Centre (MAHSC), Manchester, UK; 2 Greater Manchester Mental Health NHS Foundation Trust, Manchester, UK; 3 Centre for Youth Mental Health, The University of Melbourne, Parkville, Victoria Australia

**Keywords:** Health professional, Cardiovascular, Risk factors, Lifestyle, Professional practice

## Abstract

**Background:**

People with schizophrenia have a higher premature mortality risk compared with the general population mainly due to cardiovascular disease (CVD). Despite this, people with schizophrenia are less likely to access physical health services or have their physical health investigated and monitored.

**Aims:**

To examine the beliefs and actions of mental health professionals regarding the physical health of people with schizophrenia.

**Method:**

Two hundred and fifty-five healthcare professionals who support people with schizophrenia within Greater Manchester Mental Health NHS Foundation Trust (GMMH), United Kingdom and Pennine Care NHS Foundation Trust (PCFT), United Kingdom took part. Beliefs and actions were assessed using a self-administered questionnaire, which was constructed around two primary domains (1) CVD risk factors; and (2) physical health interventions. Descriptive statistics were reported and responses between different healthcare professional groups were compared.

**Results:**

The overwhelming majority of participants were aware of established CVD risk factors with 98% identifying family history of CVD, 98% for smoking and 96% for high blood pressure. Most participants believed nearly all healthcare professionals were responsible for monitoring the physical health of people with schizophrenia, regardless of job speciality. There were 67% of participants who reported delivering an intervention to improve sedentary behaviour for people with schizophrenia. However, awareness of government and NHS recommended lifestyle interventions were low.

**Conclusions:**

This study found good knowledge regarding many established CVD risk factors but little clarity regarding who is responsible for monitoring the physical health of people with schizophrenia and how often brief lifestyle interventions are being implemented.

## Introduction

People with schizophrenia have a reduced life expectancy by one to two decades and a premature mortality risk two to three times higher than the general population [1–3]. The most common cause of death in this group is cardiovascular disease (CVD) [4–6], with a more than double risk of death from CVD compared with the general population, according to a large-scale meta-analysis [7]. Physical health comorbidity is one cause of the poor health outcomes seen in this group, who have been shown to have high prevalence of obesity [8], type 2 diabetes [9], dyslipidaemia and hypertension [10]. Antipsychotic medication, prescribed to manage symptoms, can cause adverse side effects of metabolic syndrome [11] (defined as central obesity, plus any two of raised triglycerides, reduced high-density lipoprotein-cholesterol, raised blood pressure, raised fasting plasma glucose [12]). However, as increased risk of cardiometabolic diseases in people with schizophrenia can arise even prior to antipsychotic treatment [13], unhealthy lifestyle factors may also play a part, including increased prevalence of smoking [14], sedentary behaviour [15] and dysregulated sleep patterns [16, 17].

An added problem is that people with schizophrenia are less likely than people in the general population to report physical health problems [18–20], access health services for physical health concerns [21] and have these issues investigated and monitored [22, 23]. Lack of clarity about who is responsible for the physical health of mental health service users may contribute to this problem [24]. While primary care staff likely have the necessary skills and expertise to perform physical health assessments, knowledge and confidence may be lacking in relation to supporting people with schizophrenia [25]. A survey found 47.2% of practice nurses administer antipsychotic injections, yet only 9.4% monitor for side effects using a validated rating scale [26]. This can lead to ‘diagnostic overshadowing’ whereby treating healthcare professionals could attribute physical symptoms to an existing mental health diagnosis and subsequently do not investigate [27].

The aim of this study is therefore to examine the beliefs and actions of healthcare professionals who support people with schizophrenia, in relation to their patients’ physical health.

## Method

### Sample

We surveyed healthcare professionals who were currently working in a job role that involved contact with one or more individuals with a diagnosis of schizophrenia or related psychosis, according to the ICD-10 classification system [28]. Participants were recruited from Greater Manchester Mental Health NHS Foundation Trust (GMMH), United Kingdom (UK) and Pennine Care NHS Foundation Trust (PCFT), UK. Participants with a clinical role in supporting people with schizophrenia were identified and contacted by National Institute for Health Research (NIHR) Clinical Research Network (CRN) clinical studies officers (CSOs). The study received ethical approval from the North West Research Ethics Committee (17/NW/0368). It was also adopted by the NIHR CRN Portfolio (34859). There were 255 participants included in this study, 119 were recruited from GMMH and 136 were recruited from PCFT.

### Questionnaire development

The self-administered questionnaire was constructed around two primary domains [1] CVD risk factors; and [2] physical health interventions (Supplementary materials). The first section of the questionnaire asked about gender, job specialty, area of work, time worked in the profession and NHS trust. The second section asked participants to select on a checklist: “Which of the following do you think are risk factors for cardiovascular disease? Please tick all that apply”. Risk factors were included based on published literature [29, 30] and UK national clinical guidelines to improve health and social care by the National Institute for Health and Care Excellence (NICE) [31, 32]. Additional misleading variables, i.e. hair dye and mobile phone use, were also included as options to determine if any participants simply checked all items. Participants were excluded from the analyses if all misleading items were checked. For the purpose of this study, we considered risk factors to be established predictors of CVD if they were stated as such in NICE publications [31, 32] or used in algorithms for CVD risk prediction scores that could be applied to people with schizophrenia [33, 34].

The third section asked participants questions relating to physical health monitoring and interventions. First, participants were asked to select on a checklist which healthcare professionals they felt were responsible for monitoring the physical health of people with schizophrenia. Second, participants were asked a dichotomous question: “Have you ever delivered any effective interventions to improve sedentary behaviour in people with schizophrenia?” If participants answered “yes” they were then asked an open-ended question with a section to write comments: “If you answered yes, please specify the intervention(s)”. Finally, participants were asked dichotomous questions on whether they had heard of government recommended lifestyle interventions; Making Every Contact Count (MECC), which is an NHS approach for healthcare professionals to support lifestyle related behaviour change in any patient they come into contact with [35]; and Very Brief Advice (VBA) on smoking, which involves short and simple advice that healthcare professionals can provide opportunistically, with any patient they come into contact with who smokes [36]. An additional misleading (i.e. non-existent) intervention ‘VBA on sedentary behaviour’ was also included. If participants selected “yes” to having heard of any of these interventions, they were then asked if they had ever delivered the intervention to someone with schizophrenia.

### Questionnaire distribution

A cross-sectional online survey was distributed to participants within community mental health teams and inpatient settings using SelectSurvey.net (v4·146·001; ClassApps, Overland Park, KS, U.S.A.). Additionally, the survey was available in paper format for participants to complete and post back to the research team if they preferred. Preferred method of survey delivery was ascertained by the recruiting CSOs. All individual responses were checked for each completed survey by a researcher to ensure participants did not submit duplications via SelectSurvey.net and paper format. Participants received an information sheet to read, via SelectSurvey.net or as an attachment with the paper version. As no identifiable information was requested from participants, anonymity was preserved.

### Statistical analyses

After recruitment was completed, the survey was closed and data were downloaded from the SelectSurvey.net website and imported into Microsoft Excel software. Data were then analysed using STATA (version 13; Statacorp, TX, USA). Descriptive statistics were reported as percentages. Depending on their job role, healthcare professionals were grouped as follows: Non-qualified clinical staff, nurses, senior/managerial clinical staff, allied health professionals and medical practitioners. Specifically, the non-qualified clinical staff group comprised healthcare assistants/support workers, assistant psychologists, occupational therapy assistants, recovery/service user co-ordinators and student nurses. The nurses group comprised of mental health nurses (hospital), community psychiatric nurses, clozapine nurses and research nurses. The allied health professionals group comprised psychologists, cognitive behavioural therapy (CBT) therapists, occupational therapists, social workers, pharmacists and dietitians. The medical practitioners group comprised consultant psychiatrists, junior doctors and specialty doctors. A comparison between grouped healthcare professionals and all survey responses was made using Chi-square test, or where the expected cell count was below 5, a Fisher’s exact test. Differences between the groups were considered statistically significant if the *p*-value was less than 0.05.

## Results

### Summary data

In total 255 participants completed the survey between May 2018 and September 2018, of whom 69% were female and 41.9% were nurses. Summary data for participant characteristics can be seen in Table 1.

**Table 1:** Characteristics of healthcare professionals (*N* = 255)

	Number	Percent
**NHS Trust**		
GMMH	119	46.7
PCFT	136	53.3
**Gender**		
Male	73	28.7
Female	176	69.3
**Time in profession**		
Less than 1 year	15	5.9
1–4 years, 11 months	60	23.7
5–9 years, 11 months	50	19.8
10–19 years, 11 months	72	28.5
More than 20 years	56	22.1
**Job role**		
Non-qualified clinical staff	69	27.3
Nurses	106	41.9
Senior/managerial clinical staff	14	5.5
Allied health professionals	42	16.6
Medical practitioners	22	8.7
**Workplace**		
Inpatient care	115	45.3
Community mental health team	68	26.8
Early intervention in psychosis	30	11.8
Rehabilitation	15	5.9
Substance misuse services	4	1.6
Crisis resolution	6	2.4
A day unit	4	1.6
Other community services	12	4.7

### Risk factors for CVD

Almost all participants identified family history of CVD (98%), smoking (98%) and high blood pressure (96%) as risk factors for CVD. About three-quarters of participants identified age and antipsychotic/antidepressant medication as a risk factor. Diabetes was identified as a risk factor for CVD by 72%, ethnicity by 55% and sleep problems by 45% of participants. Table 2 shows the findings from the full list of risk factors that were available for participants to select. Among the risk factors presented, Chi-square analyses revealed significant differences between healthcare professional groups (non-qualified clinical staff, nurses, senior/managerial clinical staff, allied health professionals and medical practitioners) in whether they believed that ethnicity, diabetes and antipsychotic/antidepressant medication are risk factors for CVD (Table 3). Compared with other groups, non-qualified clinical staff were significantly less likely to identify ethnicity (31.9% vs range: 57.1–77.3%, χ^2^ = 21.24; *p* < 0.001), diabetes (60.9% vs range: 70.8–90.9%, χ^2^ = 9.77; *p* = 0.04) and antipsychotic/antidepressant medication (60.9% vs range: 71.4–90.9%; χ^2^ = 10.60; *p* = 0.03) as CVD risk factors (Table 3).

**Table 2:** Summary of risk factors identified by professional groups for CVD (N = 255)

Risk factors	Non-qualified clinical staff N (%)	Nurses N (%)	Senior/managerial clinical staff N (%)	Allied health professionals N (%)	Medical practitioners N (%)	Total N (%)
^ab^Family history of CVD	68 (98.6)	103 (97.2)	13 (92.9)	42 (100)	22 (100)	250 (98.0)
^ab^Smoking	66 (95.7)	104 (98.1)	14 (100)	41 (97.6)	22 (100)	249 (97.7)
^ab^High blood pressure	64 (92.8)	103 (97.2)	14 (100)	40 (95.2)	22 (100)	245 (96.1)
^ab^Obesity	63 (91.3)	102 (96.2)	11 (78.6)	40 (95.2)	22 (100)	240 (94.1)
^ab^High cholesterol	63 (91.3)	97 (91.5)	14 (100)	41 (97.6)	22 (100)	239 (93.7)
^a^Lack of physical activity	64 (92.8)	96 (90.1)	14 (100)	39 (92.9)	21 (95.5)	236 (92.6)
^b^Alcohol consumption	60 (87.0)	93 (87.7)	11 (78.6)	32 (76.2)	18 (81.8)	216 (84.7)
^c^Poor diet	54 (78.3)	93 (87.7)	12 (85.7)	32 (76.2)	20 (90.9)	212 (83.1)
^c^Substance abuse	58 (84.1)	87 (82.1)	11 (78.6)	33 (78.6)	16 (72.3)	206 (80.8)
^ab^Age	49 (71.0)	84 (79.3)	10 (71.4)	30 (71.4)	18 (81.8)	193 (75.7)
^ab^Antipsychotic/antidepressant medication	42 (60.9)	83 (78.3)	10 (71.4)	32 (76.2)	20 (90.9)	189 (74.1)
^ab^Diagnosis of type 2 diabetes	42 (60.9)	75 (70.8)	10 (71.4)	34 (81.0)	20 (90.9)	183 (71.8)
Other psychiatric medication	32 (46.4)	70 (66.0)	10 (71.4)	24 (57.1)	12 (54.6)	150 (58.8)
^ab^Ethnicity	22 (31.9)	64 (60.4)	8 (57.1)	26 (61.9)	17 (77.3)	139 (54.5)
Red meat consumption	36 (52.2)	49 (46.2)	7 (50.0)	23 (54.8)	11 (50.0)	128 (50.2)
Caffeine consumption	34 (49.3)	51 (48.1)	8 (57.1)	23 (54.8)	5 (22.7)	122 (47.8)
^c^Poor sleep	33 (47.8)	44 (41.5)	5 (35.7)	19 (45.2)	12 (54.6)	114 (44.7)
Dehydration	25 (36.2)	34 (32.1)	6 (42.9)	17 (40.5)	3 (13.6)	86 (33.7)
Lack of access to green spaces	19 (27.5)	30 (28.3)	2 (14.3)	12 (28.6)	7 (31.8)	71 (27.8)
^c^Air pollution	19 (27.5)	22 (22.6)	3 (21.4)	13 (31.0)	3 (13.6)	63 (24.7)
Noise pollution	5 (7.3)	10 (9.4)	1 (7.1)	6 (14.3)	1 (4.6)	23 (9.0)
Vaccinations	5 (7.3)	4 (3.8)	0	1 (2.4)	1 (4.6)	11 (4.3)
Hair dye	3 (4.4)	3 (2.8)	0	0	0	6 (2.4)
Mobile phone use	2 (2.9)	3 (2.8)	0	1 (2.4)	0	6 (2.4)

^a^Established risk factors according to NICE; ^b^Established risk factors according to CVD risk prediction scores applicable to people with schizophrenia [33, 34]; ^c^Not an established CVD risk factor according to NICE, but there is some evidence for it increasing CVD risk in published literature

**Table 3:** Comparison between groups on beliefs of risk factors for CVD

	Ethnicity		Diabetes		Antipsychotic/antidepressant medication	
	N	%	N	%	N	%
**Non-qualified clinical staff**	22	31.9	42	60.9	42	60.9
**Nurses**	64	60.4	75	70.8	83	78.3
**Senior/managerial clinical staff**	8	57.1	10	71.4	10	71.4
**Allied health professionals**	26	61.9	34	81.0	32	76.2
**Medical practitioners**	17	77.3	20	90.9	20	90.9
**χ**^**2**^	21.239		^a^9.772		^a^10.601	
***P***	< 0.001		0.04		0.03	

^a^Fisher’s exact was used due to some cell counts being < 5

### Physical health monitoring

#### Responsibility

**Fig. 1:**
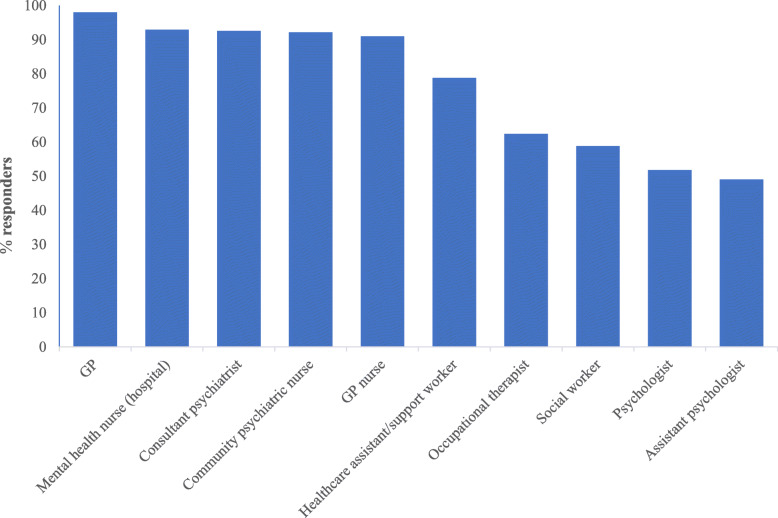
Whose responsibility is it to monitor the physical health of people with schizophrenia?

Similar numbers of participants believed the responsibility to monitor the physical health of schizophrenia was that of general practitioners (GPs) (93%), mental health nurses (hospital) (93%), consultant psychiatrists (93%), community psychiatric nurses (92%) and GP nurses (91%) (Fig. 1). There was no significant difference between groups regarding perceptions of who had responsibility for physical healthcare.

#### Interventions

**Fig. 2:**
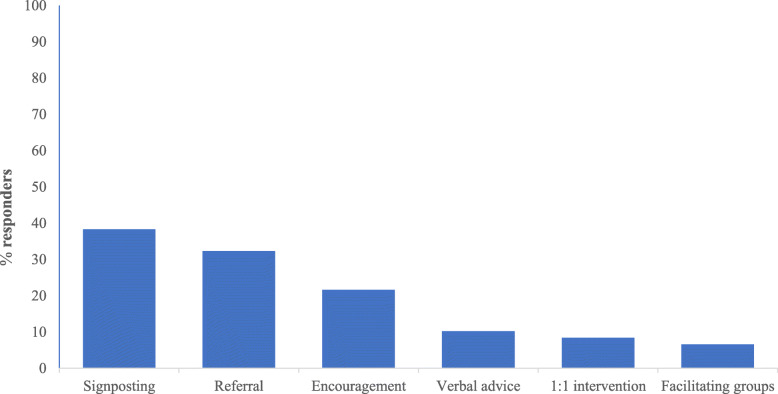
Interventions for improving sedentary behaviour in people with schizophrenia

In total 67% of participants said at some point they had delivered an effective intervention to improve sedentary behaviour in people with schizophrenia and groups differed significantly (χ^2^ = 28.67; *p* < 0.001). The group with the highest proportion of participants to answer yes to this question was nurses (83.5%, *n* = 86, 34% of the total). The most common intervention across all groups was signposting, i.e. directing patients to services or sources of advice (38%, *n* = 64, 25% of the total) (Fig. 2).

In regards to government recommended interventions, 28% (*n* = 70) of participants had heard of MECC, of whom 42% (*n* = 26, 10% of the total) stated they had used this intervention with someone with schizophrenia. 38% (*n* = 97) of participants had heard of Very Brief Advice (VBA) on smoking, of whom 60% (*n* = 56, 22% of the total) stated they had delivered this intervention to someone with schizophrenia. 13% of participants (*n* = 33) believed they had heard of a non-existent intervention, VBA on sedentary behaviour, of whom 68% (*n* = 21, 8% of the total sample) stated they had delivered this intervention to someone with schizophrenia. There was no significant difference between groups in whether they had heard of MECC, VBA on smoking or the non-existent intervention VBA on sedentary behaviour. Nor did groups differ in whether they had delivered these interventions to someone with schizophrenia.

## Discussion

Knowledge of many established risk factors for CVD was high, including some risk factors of particular relevance to schizophrenia patients, such as smoking, obesity and lack of physical activity. Other established risk factors of age, diabetes and ethnicity were less widely known. There was an educational gradient in recognition of diabetes, ethnicity and antipsychotic/antidepressant medication as risk factors, with more highly qualified professionals more likely to correctly identify these risk factors. Almost 75% of participants identified antipsychotic/antidepressant medication as a risk factor for CVD. Of concern was that nearly 10% of medical practitioners and over 20% of nurses were unaware that antipsychotic/antidepressant medication are risk factors for CVD. Antipsychotic use is stated to increase CVD risk in NICE guidance [32]. Antidepressant use was found to be associated with increased risk of CVD, independent of traditional risk factors in a CVD risk prediction model for people with severe mental illness (SMI) [34]. As well as cardiovascular side effects, antipsychotic treatment is associated with metabolic syndrome [11]. These side effects can lead to non-adherence [37], which is associated with increased use of healthcare resources [38] and reduced quality of life [39]. However, clinicians may experience reluctance to make changes to medication regimens, despite health-related risks, as pharmacological treatment for schizophrenia is often effective at treating psychotic symptoms [40], as well as reducing adverse outcomes such as relapse and hospitalisation [41].

A high proportion of participants across all professional groups selected substance abuse as a risk factor for CVD (overall 81% of the whole sample). Studies from the general population have shown recreational drugs such as methamphetamine and cocaine can provoke a multifactorial dysfunction of the cardiovascular system [42, 43]. Prevalence of substance abuse is high in people with schizophrenia [44], whereby between 40 and 50% of people with schizophrenia may have a comorbid substance use disorder (SUD) [45]. This rate is about three times as high as that of the general population [46]. SUDs are associated with poor treatment adherence and response, increase symptoms and the risk of relapse [46], and are associated with poorer physical health outcomes [47]. Substance abuse is not cited as a CVD risk factor in NICE guidance on CVD risk assessment and prevention [31, 32], although NICE does recommend monitoring the physical health of adults and young people with psychosis and comorbid substance misuse [48]. It was therefore reasonable for participants to identify substance use as a risk factor for CVD. A 2019 *Lancet Psychiatry* Commission has highlighted that people with SMI and SUD are at an increased risk of physical multimorbidity and addressing substance misuse within mental health services should be a high priority [49]. However, access to secondary health care for people with mental illness may be limited due to issues such as stigma and fragmentation of care [49]. Subsequently, people with mental illness and comorbid physical health problems likely receive an inferior standard of health care compared with the general population with the same physical health problems [49]. There is therefore a need for investment in integration of physical and mental health care.

Sleep problems have been linked to CVD in the general population [29, 50, 51]. However, they have not been included as such in NICE guidelines. Sleep problems are highly prevalent in people with schizophrenia [16, 52] but the link between sleep problems and CVD in schizophrenia requires more research. It is therefore not surprising that less than half of participants endorsed sleep as a CVD risk factor. If further research establishes sleep problems as a risk factor for CVD in schizophrenia, the assessment of sleep outcomes such as sleep quality and sleep duration should become part of routine training for NHS staff that support people with schizophrenia.

When examining whose responsibility participants felt it was to monitor the physical health of people with schizophrenia, there were high levels (over 90%) seen on half the given options and there was no difference in responses between groups. This raises a risk of “diffusion of responsibility”, in that the presence of other healthcare professionals providing support may make some feel less personally responsible for monitoring physical health [53]. Healthcare professionals in secondary care may believe the responsibility lies with healthcare professionals in primary care and vice visa.

Comparisons between groups showed significant differences in which healthcare professionals have delivered interventions to improve sedentary behaviour in people with schizophrenia, with nurses being the group with the highest proportion. This suggests that some physical health interventions are being incorporated into healthcare professionals’ patient consultations but perhaps in an informal format. There was poor awareness of either of the government recommended interventions, MECC and VBA on smoking. While both MECC and VBA on smoking are designed to be used during any patient interaction, it appears they are not being implemented. Further, this survey may have over-estimated awareness of MECC and VBA on smoking as participants may have responded ‘yes’ to all nominated interventions without actually having heard of them, as suggested by the finding that 33 participants were aware of the non-existent VBA on sedentary behaviour.

Some limitations should be considered in the interpretation of the findings of this study. Participants were asked whether they had ever delivered any effective interventions to improve sedentary behaviour in people with schizophrenia and signposting and referrals were provided as examples. First, the term ‘effective’ was not defined and it is therefore possible participants might have delivered an intervention, with no way of knowing whether it was effective. Second, it was not surprising that signposting and referrals were the two most common interventions cited given these were examples provided. This could therefore have been construed as suggestive. Nonetheless, participants did cite other interventions independently such as encouragement and verbal advice.

A strength of this study was the large number of participants across a range of clinical staff. The anonymity of the survey also enabled participants to be honest in their responses.

### Conclusions

Healthcare professionals’ awareness of many established risk factors for CVD is high but, only moderate numbers identified sleep problems or antipsychotic/antidepressant use as risk factors, with higher numbers identifying substance abuse despite this not appearing as a CVD risk factor in NICE guidance [31, 32]. Previous research found that the physical health of people with schizophrenia and related psychotic illnesses was not well monitored or investigated [54]. While we did not ask specifically about whether participants monitored patients’ physical health and risk factors for CVD, the lack of awareness about some risk factors could mean that monitoring of physical health is sub-optimal. Coupled with the lack of clarity about responsibility for physical health care, this raises the possibility that some risk factors may not be monitored and that an overall picture of a patient’s CVD risk may not be considered. Poor physical health in people with SMI decreases well-being [39], reduces adherence to prescribed medication [37] and hinders recovery from mental health symptoms [38]. This adds to the social and economic burden of their mental illness [55, 56]. We support the findings from the 2019 *Lancet Psychiatry* Commission that integrated care models are needed, with clear lines of accountability and responsibility, and good communication between primary and secondary care services [49].

## Supplementary information

**Additional file 1.**

## Data Availability

The dataset used and/or analysed during the current study are available from the corresponding author on reasonable request.

## Abbreviations

CBT: Cognitive behavioural therapy
CRN: Clinical Research Network
CSO: Clinical studies officer
CVD: Cardiovascular disease
GMMH: Greater Manchester Mental Health NHS Foundation Trust
GP: General practitioner
MECC: Making Every Contact Count
NICE: National Institute for Health and Care Excellence
NIHR: National Institute for Health Research
PCFT: Pennine Care NHS Foundation Trust
SMI: Severe mental illness
SUD: Substance use disorder
UK: United Kingdom
VBA: Very Brief Advice
