# Supplementary material for: Examining healthcare professionals’ beliefs and actions regarding the physical health of people with schizophrenia
Source: BMC Health Serv Res. 2020 Aug 20;20:771. doi: 10.1186/s12913-020-05654-z (PMC7441685; doi:10.1186/s12913-020-05654-z)
Supplement: Supplementary file 1 — Additional file 1. [file 12913_2020_5654_MOESM1_ESM.docx]

# Physical health monitoring of people with schizophrenia

REC ref: 17/NW/0368

Chief Investigator: Alexandra Berry, University of Manchester

Please answer the following questions and return to:

Miss Alexandra Berry

Room 3.306 Jean McFarlane Building

University of Manchester

Oxford Road

Manchester

M13 9PL

## By completing this questionnaire, you are confirming that:

- You have read and understood the Staff Participant Information Sheet dated 26.03.2018 (Version 1.0) for the above study, and have had the opportunity to consider the information.
- You have had the opportunity to ask questions about the study and that these questions have been answered satisfactorily.
- You are aged 16 years or older
- You understand that the data collected may be published as part of a research project. Your identity will not be revealed in any publication.

## 1. What is your gender identity?

| Male | Female |
| --- | --- |
| Prefer not to say | Other (please specify)  ………………………………………………………………………………… |

## 2. Which NHS trust do you work for?

| Greater Manchester Mental Health NHS Foundation Trust | Pennine Care NHS Foundation Trust |
| --- | --- |
| Other (please specify) ………………………………………………………………………………………………… | |

## 3. What is your job role?

| Community psychiatric nurse | GP |
| --- | --- |
| Mental health nurse (hospital) | GP nurse |
| Consultant psychiatrist | Psychologist |
| Occupational therapist | Assistant psychologist |
| Healthcare assistant / support worker | Social worker |
| Other (please specify) …………………………………………………………………………………………………. | |

## 4. Where do you work?

| Crisis resolution & home treatment | Early intervention in psychosis |
| --- | --- |
| Assertive outreach | Inpatient care |
| Community mental health team | GP surgery |
| Other (please specify) …………………………………………………………………………………………………. | |

## 5. Which of the following do you think are risk factors for cardiovascular disease? Please tick all that apply.

| Family history of cardiovascular disease | High blood pressure |
| --- | --- |
| Smoking | Caffeine consumption |
| Age | Lack of physical activity |
| Alcohol | Dehydration |
| Substance misuse | Air pollution |
| Vaccinations | Noise pollution |
| Red meat consumption | Ethnicity |
| Poor sleep | Mobile phone use |
| Obesity | Poor diet |
| Anti-depressant / antipsychotic medication | Lack of access to green space |
| Other psychiatric medication | Hair dye |
| High cholesterol | Diabetes |
| Other (please specify) …………………………………………………………………………………………………. | |

## 6. Who’s responsibility do you think it is to monitor the physical health of people with schizophrenia? Please tick all that apply.

| Consultant psychiatrist | Healthcare assistant / support worker |
| --- | --- |
| GP | Occupational therapist |
| GP nurse | Psychologist |
| Community psychiatric nurse | Assistant psychologist |
| Mental health nurse (hospital) | Social worker |
| Other (please specify) …………………………………………………………………………………………………. | |

## 7. Have you ever delivered any effective interventions to improve sedentary behaviour in people with schizophrenia? (Note. This could include signposting or referrals).

| Yes | No |
| --- | --- |

If you answered yes, please specify the intervention(s)

## 8. Have you ever heard of Making Every Contact Count (MECC)?

| Yes | No |
| --- | --- |

If you answered yes, have you ever used this intervention with someone with schizophrenia?

## 9. Have you ever heard of Very Brief Advice (VBA) on smoking?

| Yes | No |
| --- | --- |

If you answered yes, have you ever delivered this to someone with schizophrenia?

## 10. Have you ever heard of Very Brief Advice (VBA) on sedentary behaviour?

| Yes | No |
| --- | --- |

If you answered yes, have you ever delivered this to someone with schizophrenia?

## 11. How long have you been working in your profession?

| Less than 1 year | 10 years – 19 years and 11 months |
| --- | --- |
| 1 – 4 years and 11 months | More than 20 years |
| 5 years – 9 years and 11 months |  |
